# Online Training in Accessibility and Design for All: A Tool to Train Post-COVID Inclusive Graduates

**DOI:** 10.3390/ijerph182312582

**Published:** 2021-11-29

**Authors:** Ricardo Moreno-Rodriguez, Miriam Diaz-Vega, Jose Luis Lopez-Bastias, Rosa Espada-Chavarria

**Affiliations:** Education Sciences Department, Universidad Rey Juan Carlos, 28032 Madrid, Spain; miriam.diaz@urjc.es (M.D.-V.); joseluis.lopez@urjc.es (J.L.L.-B.); rosa.espada@urjc.es (R.E.-C.)

**Keywords:** sustainability, inclusion, higher education, disability, Design for All

## Abstract

Agenda 2030 expresses, through the Sustainable Development Goals (SDG), and in particular through No. 4, the need to ensure an inclusive and equitable education, which promotes learning opportunities for all. At the university level, all students are urged to acquire the necessary theoretical and practical knowledge to promote sustainable development, so that they become graduates capable of facing the challenges of the future and the real demands of a society marked by heterogeneity, including the needs of people with some kind of disability. In this sense, the present work analyzed the impact of a transversal training program in Design for All on university degree students. For this purpose, a descriptive and comparative ex post facto study was developed in which the impact of an online training program was quantified by establishing comparative pre- and post-training. The results indicate that the approach, through the delivery of a training xplain eon Design for All, contributed to a change in the perceptions of students regarding disability, its role in the university and in the future workplace. Furthermore, it increased the knowledge of institutional action undertaken in terms of awareness and approach to human disability.

## 1. Introduction

Agenda 2030 [1,2] gives a primordial role to education as a tool to promote sustainable development. In addition, its relevance is highlighted by offering one of the objectives (number 4) to guarantee an inclusive and equitable education, which can promote opportunities for lifelong learning for all. Furthermore, at the same time, by guaranteeing this goal it is promoting training and global learning in the other 16 objectives [3].

The aim of SDG 4 is to complete the process that began with the Millennium Goals [4] and that remained unfinished, as reflected in the *Final Report of the United Nations Decade of Education for Sustainable Development* [5]. This report emphasizes the progress that must be done in training in sustainability (environmental dimension) just because serious deficiencies had been detected in Human Rights and in the Rights of People with Disabilities (social dimension of Human Development).

The same lack is detected regarding to the most important university documents in Spain: “Cómo evaluar los ODS en las Universidades” [6] and “Implementando la Agenda 2030 en la Universidad. Casos inspiradores” [7]. In both of them, activity and university assessment is oriented almost exclusively to the field of environmental management and education, observing a lack in its orientation toward the training about human rights, which must be necessarily addressed for the achievement of Agenda 2030 [1,8,9,10].

From SDG 4, the need to guarantee the access, participation and achievement of education for all people has been raised at the same time that equity and equality of opportunities have to guide the principles of education at all levels [1].

Similarly, and focusing on higher education, target seven of SDG 4 [2] asks universities to ensure that, by 2030, all students must acquire the knowledge and necessary skills to promote sustainable development goals, so that university students can turn into graduates capable of facing the challenges of the future and the real demands of a society, marked by heterogeneity. In this sense, it is expressly alluding to the development of training and skills centered on human rights that allow the needs of people with disability to be covered [1].

These objectives are neither new nor leading edge. In Europe, following the creation of the European Higher Education Area (EHEA) and with the signing of the Bologna Declaration by the Ministers of Education in June 1999, a turning point was generated which made it possible to propose substantive changes in the educational and management model of European universities.

On the other hand, the conception of disability is embodied in the Convention on the Rights of Persons with Disabilities—UNCRPD (UN, 2006)—as a normative response, where medical and social models converge, and explaining disability as a factor within society and not within the person, making it an issue that directly affects human rights (Ospina, 2010). The Convention was ratified by Spain in 2008, being one of the first countries to do so. Based on this approach, persons with disabilities are no longer considered as objects of assistance or charity, but as subjects of rights and obligations.

UNCRPD makes it mandatory to examine the situation of each person with a disability from his or her personal, social and/or cultural context, giving special attention to the elements of the environment and the individual. It therefore becomes a legally binding and enforceable instrument that recognizes social justice, equal rights, equity, belonging, social inclusion and equal opportunities, giving legal status to the concept of disability.

Under the slogan “no one is left behind” included in SDG 10, the importance given to the role of education for development and for the generation of future values that allow the achievement of the goals as a whole is highlighted, with special mention in SDG 4 to inclusive quality education [9].

For its part, SDG 4 proposes that the specific educational needs of people with disabilities be addressed at all educational levels, in order to guarantee participation in this basic need, reduce inequalities and increase development opportunities, transferring to this goal the tools defined by the UNCRPD (universal accessibility, universal design for all and reasonable adjustments) as well as the articles referring to the right to education, where the UNCRPD recognizes the right to inclusive education and to be educated in the same schools as everyone else.

It is assumed that inclusive education values diversity as an aspect that enriches the teaching–learning process and favors human development, and the UNCRPD states that the guarantee of the right to education (not only at the initial and compulsory levels, but also at the university level) is the right to learn in an inclusive education system that not only provides quality education, but also changes discriminatory attitudes and discriminatory systems to create inclusive societies that respect and value, in terms of equality, the differences and dignity of individuals.

Training of future professionals from a competence perspective becomes the cornerstone of university degrees, despite criticism of the implementation of this model, in which there is a perceived proximity to the requirements of the market to the detriment of the humanist essence considered important to address human development [11]. The main objective of this model of the university system is to provide graduates with specific tools that allow them to face the demands of society by their application [12].

Advancing toward an improvement in European education, the EHEA social dimension [13] was born, with the aims to ensure equal opportunities in access, participation and success for all studies in all areas covered by the university system [14], which is reinforced by the approval of the Europe Strategy 2010–2020, and which calls for the incorporation of specific training in all degree courses by integrating Universal Design and Design for All.

This scenario reinforces and supports the fundamental role that universities have to face the challenges of the future, not only from the training and research point of view, but also from relationship that must be established between the educational institution and the society in which it is immersed, highlighting the transforming essence of education itself and assuming it as a right that enables other rights [1].

From this perspective, various studies analyze and affirm the capacity that the university has to disseminate and put into practice the set of values and principles that define this responsibility through its foundational functions (management, teaching, research and university extension) [15,16,17,18,19,20,21,22,23]. Furthermore, Spain has to approach it with the ethical commitment acquired as a member of the United Nations but also with the legal framework that promotes the rights of people with disabilities and their social inclusion [24].

In Spain, Article 3.5b of Royal Decree 1393/2007 of 29 October on Graduate Degrees establishes the general principles that must inspire the design of the degrees, emphasizing that all degrees must contribute to the knowledge of the principles of accessibility and design for all people, with the aim of ensuring training that guarantees university graduates. Regardless of the professional profile or field of knowledge to which their degree belongs, they will be able to apply the principles of Universal Design in their future profession, as stated in Royal Legislative Decree 1/2013, of 29 November, which approved the Revised Text of the General Law on the Rights of Persons with Disabilities and Their Social Inclusion.

Four years after the approval of this Royal Decree, the results of various studies which analyze the implementation of this norm in university degrees were published in Spain. The University and Disability Observatory [25] presents the results obtained after analyzing the syllabus of 23 universities in Galicia, the Valencian Community, Andalusia, Castilla la Mancha, Castilla León and Extremadura. The results highlight a low presence of the contents searched for in all the fields of knowledge.

In this line, the study developed by the Association for Community Solidarity of People with Functional Diversity and Social Inclusion [26], analyzed the inclusion of universal accessibility and design for all people in 76 Spanish universities, and indicated that only 10% of them (seven in total) fully comply with this standard.

Iglesias, Saraiva and Lloredo [27] analyze the state of this issue in the same 76 universities, identifying the subjects that developed accessibility-related content in different degree courses. In this case, the percentage of Spanish universities that complied with the standard was 16%, far from the desired situation.

Apart from that, Sánchez and Díez [28] analyzed 159 teaching guides for degrees related to computer engineering and architecture. Their results coincide with the low level of compliance with the standard detected in 90% of Spanish universities.

Universia Foundation [29], in its IV Study on University and Disability, elaborated from the data offered by the services of attention to people with disability at the university level [30], exposed that only 14.9% of the 50 Spanish universities answered affirmatively when asked about the inclusion of the disability variable in the degree programs. Moreover, 20.3% of the services indicated that it was reflected in some of them, without specifying which ones and to what extent; 16.2% answered in a negative way and more than 40% of the services indicated “no record”.

The most recent study that analyzes the inclusion of Universal Design and Design for All people in university degrees, related to engineering, architecture, design and psychology, reflects a hopeful improvement and awareness of the need to contemplate and comply with current regulations, but reveals that the real level of depth in university education is still insufficient and reveals in most cases a lack of commitment by the government board of Spanish universities [31]

Once this training gap was detected, it was possible to identify the specific training needs in this area, owing to the contributions of various authors [32,33,34,35,36] who analyzed the attitudes of professionals, teachers and students toward people with disabilities, by using scales designed by different authors [37,38,39,40,41]. These studies have been a very valuable source of information to identify training deficiencies, specific information needs and to eliminate false beliefs and stereotypes toward the group of people with disabilities.

Based on these data, and supported by the *White Paper on Design for All in the University* (Imserso, ONCE Foundation and Coordinator of Design for All in Spain, 2006), Rey Juan Carlos University implements mandatory cross-disciplinary training for all undergraduate students (Unit for Attention to People with Disabilities and Special Educational Needs, 2012), with the aim of guaranteeing compliance with the current regulations in force and thus enabling the promotion of training in sustainable development for all university students, thus generating graduates committed to diversity and disability.

The design and presentation of this training is based on the use of technology as a tool that favors the learning process, making use of its potential as a support element for access to training and information, in line with the precepts of Universal Design represented by Hall, Cohen, Vue and Ganley [42] and Meyer, Rose and Gordon [43].

For all this, the objective of the present work is to analyze the impact of transversal training related to universal accessibility and design for all people in the students of university degrees.

## 2. Materials and Methods

An ex post facto descriptive and comparative study was developed in which the impact of an online training program was quantified by establishing a comparative pre- and post-training evaluation. Before starting the specific training, the enrolled students had to fill in the ad hoc questionnaire offered. Once completed, they had access to the materials designed as a learning element, called Universal Accessibility and Design for All, displayed through the online platform of Rey Juan Carlos University. Once the training was completed and the learning assessment process was passed, the student was asked to fill out the ad hoc questionnaire again (post-training evaluation). The results were analyzed with Statistical Package for the Social Science (SPSS v22) by comparing the answers through the application of the Student t-test of paired samples and the formulation of ANOVAs for the contrast of the variances in the post-measurement.

### Description of the Training Program

The subject is presented in an online format and is hosted in the online platform of Rey Juan Carlos University. This training is part of the Academic Recognition of Credits (a compulsory multidisciplinary subject that is part of the training curriculum for all degrees programs) and is called Universal Accessibility and Design for All.

The main objectives of this training program are [44]:

- To become aware of the needs of people with disabilities, equal opportunities and universal accessibility.
- To know the general criteria of design for all and its application to professional practice.
- To assimilate the importance of incorporating criteria in professional practice that allows any person, regardless of their abilities, to enjoy all products or services.
- To make the future professional aware of the implications of universal accessibility and design for all people in independent living.

These objectives are approached from a program of selected contents designed to be of interest and applicable to the whole student body. The training program is structured in two different parts. The first is focused on transmitting a solid concept of disability and inclusion. During the second part, the barriers that hinder such inclusion and the tools that must be implemented to eliminate them and guarantee full participation of all the people that make up society are addressed in depth. Contents are:

1. General principles on functionality, capacity, disability and inclusion.
  - Concepts of disability and inclusion.
  - Main needs of people with disabilities.
2. Environment control, Universal Accessibility and Design for All.
  - What is anthropometry?
  - Support products and adaptations for people with disabilities.
  - Accessibility to the physical environment.
  - Accessibility to information.
  - Cognitive accessibility.
  - Principles of Design for All.

Once they have accessed and analyzed the material offered, two actions are proposed that serve as elements of evaluation: an online exam composed of 30 multiple choice questions and the elaboration of a personal reflection applying the precepts of Universal Accessibility and Design for All to their future profession. Only when they have passed this evaluation are they given access to the questionnaire on which this study is based.

The study was developed with 1479 students enrolled in undergraduate degrees during the 2019–2020 academic year in the subject “Universal Accessibility and Design for All People” at Rey Juan Carlos University. This figure corresponds to 100% of the available population, being the total number of students who completed this subject during the second semester of the specified academic year, so the size guarantees representativeness [45]. The sampling, therefore, is intentional, given that it was a requirement that the participants meet identical characteristics (be able to take the subject linked to academic recognition of credits), there was no randomization of participants; all were enrolled in the training program at the time of data collection.

An ad hoc questionnaire was administered to the sample. It identifies, firstly, the academic profile and gender of the participants and, secondly, it offers ten items (Table 1) organized in a Likert-type scale (from 1 “totally disagree” to 5 “totally agree”) that allowed us to know their perception about the level of knowledge acquired in Universal Accessibility and Design for All after having completed the training proposal.

Delphi methodology was used to construct the final version of questionnaire. The collaboration of different profiles related to the type of research program was requested: a psychologist with extensive training and professional experience in units of attention to students with disabilities, two occupational therapists, two teachers of primary education specialized in care for special educational needs and a special education teacher with extensive experience and professional experience in the field of care for students with disabilities. All of them served as a panel of experts to confirm the content validity of the items that make up the questionnaire.

Three rounds of anonymous consultation were carried out, each separated by a time interval of 15 days. Initially, each of the experts was given a description of the training program and all the elements to be measured with the items proposed in the questionnaire. During the validation process, each of the experts received the exam that would be used to measure the knowledge acquired in Universal Accessibility and Design for All.

They also received a table with all the proposed items from the questionnaire with the objective of evaluating the suitability, importance and observability of each one of them using a Likert-type scale from 1 to 4, with 1 being “totally disagree”, 2 “disagree”, 3 “agree” and 4 “totally agree”.

The items were considered valid when they obtained a score of 3 or more from 80% of the experts. Following their considerations, corrections were made to the wording of the items until the final version of the questionnaire was obtained, which, in the last round, obtained an overall average score of 3.8.

## 3. Results

The analysis of data was carried out according to the branch of knowledge of the degrees in which students were enrolled. The field of knowledge coincides with the geographical location of the participants. This geographical distribution corresponds to each of the campuses of Rey Juan Carlos University and maintains some correspondence with the field of knowledge. Thus, in Alcorcón are located Health Sciences studies; in Móstoles are located the Experimental Sciences and Technology studies; in Fuenlabrada are located Engineering and Architecture studies; in Aranjuez are located Arts and Humanities. Finally, in Madrid are located Legal and Social Sciences studies (Table 2).

The Student t-test was performed for comparison of two repeated measures in order to analyze the existence of significant differences in the pre- and post-evaluation. The statistical significance obtained is *p* = 0.000 in all items, when comparing the change produced in the answers after the follow-up of the subject.

In general, it is observed in the analysis of the frequency distribution of post-training that, in each of the items, the pre scores are modified toward the higher extremes of the score (“strongly agree” and “totally agree”) once the training is finished. Furthermore, there is no correlation or noticeable differences between men and women (reaching identical degrees of significance). Although it is true that the highest scores, both in the pre- and post-training, are given by the women in all the campuses.

Significant differences were observed after the development of the training program, obtaining different degrees of significance. These values together with their standard deviations are shown in Table 3.

As for the distribution of the frequency of answers, it is interesting to analyze how the score changed after the online training.

In the pre-training evaluation, 22.2% of the sample had little or no knowledge about disability and its involvement in the daily lives of the people who presented it; 19.9% of the students indicated that they had little or no knowledge of the different barriers that can limit the participation and autonomy of people with disabilities. This aspect is modified to positive values (between “strongly agree” and “totally agree”) after the training by representing 91.1% and 97.8% of the respective answers. In both cases, the answers related to the value “totally disagree” disappear.

When asked about knowledge of Universal Accessibility or Design for All and the principles behind them, the pre-training evaluation found that 28% of the sample knew almost nothing about Universal Accessibility and 27.9% knew little or nothing about Design for All. In addition, 11% did not agree that they understood the need to transfer these concepts to their future profession, and 59% indicated that they felt incapable of developing their profession from a design-for-all approach. Along with that, 73% of the participants felt quite incapable of working in a work team with people with disabilities.

After training, these values were notably modified, finding that 87.6% of the sample knew and understood the concept of Universal Accessibility. In addition, 74.2% knew and understood the concept of Design for All. As for the need to transfer these principles to their future profession, 41.7% agreed with this need (compared to 61% in the previous evaluation) and 58.3% of them totally agreed with that need (compared to 28% in the previous evaluation). In the post-training evaluation, 83.6% of the sample agreed totally with the idea of feeling capable of developing their profession from the design approach for all people (as opposed to 41% who moderately agreed and the cases that scored unfavorably on this issue disappeared) and 76.7% strongly agreed that they could form a teamwork with people with disabilities (along with 3.9% that totally agreed with the idea).

Concerning the statement “It is the duty of all people to create fairer and more inclusive communities and societies”, the scores were significantly modified, as can be seen in Table 4.

It could be established that there was a correlation between the modifications of the scores and the academic field of the subjects (Pearson = 0.101; *p* = 0.000). Levene’s test for homogeneity of variances indicates that the variance is not the same in all groups (in each of the fields of knowledge) for all items, except in the item “I know and understand the need to transfer these concepts to my daily life and future profession” (*p* = 0.987) where equal variances can be assumed in all groups. As a consequence, all participants have understood the implications of the training in their future work. The fact that there are different variances by areas of knowledge is related to the type of studies carried out and the ease of generalizing the contents studied to their respective professional performances.

After the completion of the single-factor ANOVA test, it can be concluded that, although significant differences have been obtained with the Student t-test, there are also differences in the acquisition of knowledge by each of the groups. Thus, there is heterogeneity of means in all the variables except in “I know and understand the concept of disability and its implication in the daily life of the people who present it” (F = 0.864; *p* = 0.485) and in “I know and understand the need to transfer these concepts to my daily life and my future profession” (F = 0.24; *p* = 0.999), where it seems that all the participants of the different groups acquired a similar and positive knowledge with respect to the pre-evaluation.

Scheffe’s post hoc test was applied to those variables in which heterogeneity was detected. In this way, in the item “I understand that the design and structure of the environment and elements that compose it are those that can limit the full participation of people with some kind of disability”, it was found that there was a significant difference (*p* = 0.048) between students of Legal and Social Sciences and those of Arts and Humanities. In this sense, lower scores in students from Legal and Social Sciences were observed (MD = −0.065).

Regarding the question “I know the different types of barriers that can limit the participation and autonomy of people with disabilities in their daily lives”, significant differences (*p* = 0.004) were obtained between the participants in Legal and Social Sciences and those from Experimental Sciences and Technologies, with the former scoring lower (MD = −0.154). The same happened with the item “I know and understand the concept of Universal Accessibility”, where the differences were grouped among the students of Legal and Social Sciences, who scored higher than those of Experimental Sciences and Technology (MD = −0.111; *p* = 0.002), and those of Engineering and Architecture (MD = −0.98; *p* = 0.002).

The same situation occurred regarding to the item “I know and understand the concept of Design for All and the seven principles that comprise it”, but this time the students of Legal and Social Sciences scored lower than those of Experimental Sciences and Technology and those of Engineering and Architecture (MD = −0.229; *p* = 0.000 and MD = −0.203; *p* = 0.000, respectively).

The item “I feel capable of developing my profession from the Design for All approach” presented differences among fields of knowledge. Thus, participants in Health Sciences scored lower than those in Experimental Sciences and Technologies (MD = −0.187; *p* = 0.014) and in Arts and Humanities (MD = −0.205; *p* = 0.021). On the other hand, students in Legal and Social Sciences scored lower than those in Experimental Sciences and Technology (MD = −0.095; *p* = 0.007) and those in Arts and Humanities (MD = −0.169; *p* = 0.019).

Concerning the question “I feel capable of being part of a team at work with people with disabilities”, students in Health Sciences scored lower than those in Engineering and Architecture (MD = −0.196; *p* = 0.000) and those in Legal and Social Sciences (MD = −0.135; *p* = 0.043). On the other hand, participants in Experimental Sciences and Technology presented lower scores than those in Arts and Humanities (MD = −0.148; *p* = 0.002).

The greatest difference was found in the following item: “I understand that people with disabilities have the same rights and obligations as any other member of the community”. Health Science participants showed lower scores than the other areas of knowledge. The other areas showed the following scores: Experimental Sciences and Technology (MD = −0.307; *p* = 0.000), Engineering and Architecture (MD = −0.24; *p* = 0.000), Legal and Social Sciences (MD = −0.332; *p* = 0.000) and Arts and Humanities (MD = −0.425; *p* = 0.000). Additionally, participants from Engineering and Architecture scored lower than those in Arts and Humanities (MD = −0.184; *p* = 0.015).

The last item, “It is the duty of all people to create more fair and inclusive communities and societies”, participants in Engineering and Architecture scored lower than those in Legal and Social Sciences (MD = −0.128; *p* = 0.007) and, almost assuming statistical significance (*p* = 0.052), it was found that students in Arts and Humanities scored lower than those in Legal and Social Sciences (MD = −0.147).

## 4. Discussion

The approach to training in sustainable development through the provision of a training module on Universal Accessibility and Design for All seems to have contributed to a change in students’ perceptions of disability, the role of disability in the university and in their future employment. Furthermore, it provided students with a better knowledge of institutional actions undertaken in terms of awareness and approach to human disability.

The importance of implementing and developing this training is seen in the high significance offered by the difference between the pre- and post-training evaluation moment, where differences can be seen in the stereotypes established about people with disabilities, so that certain stigmas (such as the one present in item 8 about being part of a team with people with disabilities) could be reduced or eliminated with the training.

Related to this, and according to López, Laviana, Fernández, López, Rodríguez and Aparicio [46], stigma is not only associated with people with mental illness, but refers to the set of attitudes, usually negative, that a social group maintains with other minority groups—a universal phenomenon that is related to the processes of social categorization.

This coincides with studies such as those carried out by Aguado, Flórez and Alcedo [47], which maintain that collective attitudes still have a pejorative component that comes from false myths and historical prejudices that make people with disabilities appear inferior, incompetent or incapable.

The differences in the homogeneity of the impact observed in the sample (detected with the Levene test and the one-factor ANOVA) can be explained by the difference in contents and in the structure of the degrees according to their field of knowledge. In this sense, a greater homogeneity was observed in the items that allude to conceptual aspects that may be common, such as disability or the professional responsibility of incorporating accessibility and design for all principles.

The training program seems to have increased the scores in terms of the attitudes shown by the group of students, similar to other studies such as [48,49], where a favorable attitude toward disability was observed, increasing as the age of the students increases. A similar feature is indicated by González and Roses [50] and Lopez-Bastias and Moreno-Rodriguez [51], who point out that, although university students show positive attitudes toward disability, these are favored by training and information, knowledge and direct experience. It cannot be forgotten that the distribution of response frequencies is oriented toward the higher scores of the Likert scale in the post-training evaluation, with the scores of the lower values disappearing.

A similar observation can be made with the results obtained with the Scheffe test, where the lower scores from the field of Health Sciences may be due to the training contents and the perspective offered, together with certain presences of the medical model that may condition the opinion of the future graduates about the aspects collected.

It is reasonable, on the other hand, that aspects such as Design for All mean lower scores for participants in Legal and Social Sciences, who are not used to incorporating these contents into their training syllabus.

Similarly, the concept of universal accessibility seems to score lower on degrees that, since its design, incorporate such content into some subjects of their syllabus. However, this may be due to the fact that the vision provided in Engineering, Architecture and Arts and Humanities refers to a single aspect of accessibility and not to the global concept (physical, cognitive and access to communication and information).

No significant gender differences were found, similar to most recent studies [36,52,53,54], reinforcing the effect of this training to improve the global scores, which is independent from gender.

Training in Universal Accessibility and Design for All, even consisting of a short module, improves knowledge about the concept of disability and the implications it has on people’s daily lives. Furthermore, it has a clear positive impact on the attitude of the participants, resulting mainly in the need to incorporate the variables of Universal Accessibility and Design for All into the performance of the future profession of students, which allows for responding to the training and attitudinal needs detected in previous studies [32,33,34,35,36,37].

Although there are significant changes in all the study variables, the greatest power of these changes seems to be directed at aspects related to ethical and professional commitment. This commitment favors changes in attitudes, which are necessary to promote human development toward the creation of more just, equitable and inclusive societies. In line with that proposed in ODS 4 [1,2,9,21], it is understood that education, from its potential to transform societies, extends this potential transversally to the other objectives that comprise Agenda 2030.

On the other hand, in a post-pandemic educational scenario where online learning environments have predominated, it was possible to verify the effectiveness of this training action, with similar characteristics to MOOC courses. COVID-19 has brought about a change in the functioning of educational institutions and, more specifically, of universities. In them, it has been possible to verify how the requirement of certain competencies became secondary when adapting education in the context of home confinement [55]. The benefits of dealing with more concrete and direct content, eliminating secondary information, was also verified, favoring significant learning in students, with knowledge that could be achieved in the short term so as not to harm their motivation for learning [56]. In this sense, this training program was already adapted to these characteristics since its implementation in 2012.

It is highly recommended that training in Universal Accessibility and Design for All should be incorporated as soon as possible into the curricular structure of the different university degrees to make an effective commitment to human development. That could bring about a strategy that promotes the fulfilment of the goals derived from the Sustainable Development Goals linked to the function of the university. This conclusion is in line with that proposed by the Universia Foundation [29] and Madrid, García and Campo [31].

## 5. Conclusions

Training in Universal Accessibility and Design for All, even consisting of a short module, improves knowledge about the concept of disability and the implications it has on people’s daily lives. Furthermore, it has a clear positive impact on the attitude of the participants, resulting in the need to incorporate the variables of Universal Accessibility and Design for All in the performance of the future profession of the students. Apart from that, this could allow for responding to the training and attitudinal needs detected in previous studies [32,33,34,35,36,37]. On the other hand, in the post-COVID educational scenario, this online training has proven to be a suitable option for the teaching–learning process that has also allowed education in values. It is a simple and efficient training model with high social impact, which allows for improving citizenship values and soft skills that are necessary to build inclusive societies.

The learnings acquired, which are directly related to human development, are strongly influenced by Amartya Sen’s capabilities approach and the influence that this approach has on the conceptualization of disability. This conceptualization, typical of the social model, describes disability as a social construction and focuses on the context as a generator of dependency. It can be argued that the capabilities approach that inspires the SDGs means that, at the same time, the 2030 Agenda is perfectly aligned with the International Convention on the Rights of Persons with Disabilities. It is precisely the concepts of accessibility and design for all people that become fundamental tools in the aspiration to build inclusive societies and to ensure inclusive education [57,58]. Additionally, the result of this training program, focused on these concepts, allows us to affirm that the university can be the necessary agent of change to meet the goals proposed by the 2030 Agenda, as it prepares professionals to pursue sustainable economic, environmental and human development.

Although there are significant changes in all study variables, the greatest power of these changes seems to be directed to aspects related to ethical and professional commitment. This commitment favors the change in attitudes necessary to promote human development toward the creation of fairer, more equitable and inclusive societies, in line with what is proposed in SDG 4 [1,2,9,21]. SDG 4 understands education from its potential to transform societies, extending this potential transversally to the other objectives of Agenda 2030. Reaching a high number of university students, from different disciplines, through this training program allows us to transfer an education in values required to ensure an inclusive, equitable and socially responsible society, which considers disability as a variable that exists in any work environment.

On the other hand, in the current educational scenario, where health measures (such as capacity restrictions or interpersonal distance) are necessary to guarantee public health, the proposal of this training model enables a large number of students to be reached simultaneously during each academic course. In addition, it allows the student to graduate the task and make decisions about their learning process, which lead to meaningful learning that allows them to relate the contents to their daily life and future professional performance.

## Figures and Tables

**Table 1:** Questionnaire’s items used for data collection and pre- and post-training comparison.

Item	Statement
1	I know and understand the concept of disability and its implication in the daily life of the people who present it.
2	I understand that the design and structure of the environment and elements that make it up are those that can limit the full participation of people with some kind of disability.
3	I know the different types of barriers that can limit the participation and autonomy of people with disabilities in their daily lives.
4	I know and I understand the concept of Universal Accessibility
5	I know and I understand the concept of Design for All and the seven principles that define it.
6	I know and understand the need to translate these concepts into my daily life and my future profession
7	I feel capable to develop my profession from the “Design for All” approach.
8	I feel capable to form a team with people with disabilities.
9	I understand that people with disabilities have the same rights and obligations as any other member of society.
10	It is the duty of all people to create fairer and more inclusive communities and societies.

**Table 2:** Distribution of responses by field of knowledge and gender.

Branch of Knowledge	Female	Male
Health Sciences	103	51
Sciences	137	135
Engineering and architecture	279	153
Social and Law Sciences	268	203
Arts and Humanities	101	49

**Table 3:** Significance level with Student t-test and standard deviations in pre- and post-training evaluation.

	SD	*p*
Item 1	0.68	0.00
Item 2	0.21	0.00
Item 3	0.48	0.00
Item 4	0.57	0.00
Item 5	0.50	0.00
Item 6	0.77	0.00
Item 7	0.72	0.00
Item 8	0.63	0.00
Item 9	0.64	0.00
Item 10	0.65	0.00

**Table 4:** Distribution of response percentages in item 10.

	Pre-Training	Post-Training
Totally disagree	1.60%	-----
Slightly agree	5.40%	-----
Agree	89.70%	6.90%
Strongly agree	3.30%	71.30%
Totally agree	-----	21.80%

## Data Availability

Contact with authors.
